# Development of a Non-Invasive Indirect ELISA for Detecting IgY Against a Novel Avian Metapneumovirus Subtype C in Egg Yolks of Laying Ducks

**DOI:** 10.3390/ani16182963

**Published:** 2026-09-20

**Authors:** Lin Lin, Shen Cao, Manman Guo, Nansong Jiang, Longfei Cheng, Hongmei Chen, Rongchang Liu, Chunhe Wan, Yu Huang, Qizhang Liang, Weiwei Wang, Guanghua Fu, Qiuling Fu

**Affiliations:** 1 Institute of Animal Husbandry and Veterinary Medicine, Fujian Academy of Agricultural Sciences, Fuzhou 350013, Chinaliurongc@foxmail.com (R.L.); qzl1181@zuaa.zju.edu.cn (Q.L.); wangweiweihn@163.com (W.W.); 2 Fujian Key Laboratory for Control and Prevention of Avian Diseases, Fuzhou 350013, China; 3 Fujian Industry Technology Innovation Research Academy of Livestock and Poultry Diseases Prevention and Control, Fuzhou 350013, China

**Keywords:** avian metapneumovirus subtype C, indirect ELISA (iELISA), Immunoglobulin Y (IgY), duck

## Abstract

Avian metapneumovirus subtype C (aMPV/C) has re-emerged as a widespread pathogen in China, causing hydrosalpinx fluid syndrome (HFS) in laying ducks and resulting in substantial economic losses, highlighting the urgent need for a reliable immunological detection method. In the present study, a non-invasive indirect enzyme-linked immunosorbent assay (iELISA) was developed and optimized for detecting aMPV/C-specific immunoglobulin Y (IgY) in egg yolks of laying ducks, using purified novel aMPV/C as the coating antigen. Checkerboard titration established the optimal conditions as an antigen concentration of 1.25 μg/mL and an IgY dilution of 1:200. The cut-off value, determined by receiver operating characteristic curve analysis, was 0.3268, enabling a detection limit of 1:3200 for IgY antibodies. The assay showed no cross-reactivity with IgY positive for other duck pathogens, and both intra-assay and inter-assay coefficients of variation were less than 10%, demonstrating good specificity and reproducibility. Clinical evaluation of 151 duck egg yolk samples yielded an overall aMPV/C-specific IgY positivity rate of 58.3% (88/151). The concordance rate between the iELISA and an indirect immunofluorescence assay (IFA) was 97.8% (45/46). These results indicate that the established iELISA for detecting aMPV/C-specific IgY is a reliable and practical tool for large-scale detection of aMPV/C exposure in clinical duck egg yolk samples.

## Simple Summary

This study addresses the urgent need for reliable immunological assays for novel avian metapneumovirus subtype C (aMPV/C), a re-emerging pathogen causing hydrosalpinx fluid syndrome and substantial economic losses in China’s laying duck industry. In the absence of licensed vaccines or antiviral drugs, rapid diagnosis and timely culling of infected ducks remain the primary control measures. We developed a cost-effective indirect ELISA (iELISA) using purified whole inactivated aMPV/C as the coating antigen. The assay demonstrated high specificity (no cross-reactivity with other major duck pathogens), excellent repeatability (CVs < 10%), and high sensitivity (detection limit of 1:200). Clinical evaluation of 151 egg yolk samples from three duck breeds revealed an overall aMPV/C-specific IgY positivity rate of 58.3%, with breed-specific rates ranging from 34.4% to 67.5%. The iELISA showed 97.8% concordance with the immunofluorescence assay (45/46 samples). This work provides a reliable, high-throughput tool for large-scale surveillance of aMPV/C in laying ducks, directly supporting disease monitoring and control efforts in the poultry industry.

## 1. Introduction

Avian metapneumovirus (aMPV) was first identified in South Africa in 1978 and has since spread globally, infecting both domestic poultry and wild avian species [1,2]. In chickens and turkeys, infection typically manifests as upper respiratory tract inflammation, swollen head syndrome, and reduced egg production [2,3]. In ducks, infections are generally mild, presenting with upper respiratory signs and coughing [4,5]. However, secondary infections can exacerbate disease severity and even result in mortality [2]. In laying breeder ducks, aMPV infection decreases egg output and is associated with the increasingly prevalent hydrosalpinx fluid syndrome (HFS) in China [6,7]. Based on antigenic and genetic heterogeneity, aMPV is classified into four subtypes (A-D) [8,9,10]. Subtypes A and B occur globally, with subtype B predominating in European and Chinese poultry [11]. Subtype C, initially isolated from turkeys, has a broad host range encompassing chickens, ducks, and wild birds. Notably, ducks exhibit higher susceptibility to subtype C than to other aMPV subtypes. This subtype is further divided into North American and Eurasian lineages [12] and shares the closest phylogenetic relationship with human metapneumovirus, suggesting potential public health implications via zoonotic risk [2]. Subtype D was exclusively detected in France [13]. Additionally, the detection of genetically divergent aMPV strains in North American wild birds in 2019, together with the emergence of subtypes A and B in US poultry since 2023, highlights the rapid genetic diversification of aMPV [14,15,16].

In China, aMPV/C was initially isolated from Muscovy ducks in 2014, with a subsequent gap in waterfowl reports until 2021 [4]. In subsequent years, it became increasingly prevalent, contributing substantially to reduced egg production and the occurrence of HFS in breeding and laying duck flocks throughout several provinces [5,6]. More recently, a novel aMPV/C lineage, designated aMPV-FJ21, was isolated from Sheldrake ducks exhibiting HFS [7]. Genomic characterization demonstrated that this novel variant is genetically distinct from both North American and Eurasian lineages, indicating that the virus continues to evolve and adapt to more domestic duck hosts, thereby underscoring the pressing need for effective control measures, especially rapid diagnostic tools [7].

Although clinical signs and gross pathological changes may provide preliminary diagnostic clues for HFS, laboratory confirmation remains essential. Currently, a range of diagnostic modalities are employed for aMPV/C detection, including virological isolation, molecular testing, and serological approaches. Virus isolation is labor-intensive and has low recovery rates (~8%), limiting its clinical utility [4,17,18]. Molecular methods, despite high sensitivity and specificity, are hindered by the narrow detection window of aMPV/C (typically 2–10 days post-infection) and sampling stress in laying ducks [19,20]. Unlike viral RNA, which is detectable only transiently during the acute phase, antibody responses persist for a considerably longer period: aMPV/C-specific antibodies generally become detectable in serum at approximately 7–10 days post-infection and are efficiently transferred into egg yolk, where IgY can remain detectable for several weeks to months after infection, with yolk IgY detection lagging slightly behind serum seroconversion [21,22]. This extended detection window, together with the non-invasive and convenient nature of egg yolk sampling, renders IgY-based serological assays particularly suitable for large-scale and retrospective surveillance of aMPV/C infection [23]. These limitations reduce their suitability for routine surveillance and highlight the need for serological assays [24]. ELISA is the gold standard for serological detection, offering high throughput, specificity, and sensitivity [25,26]. However, no commercial ELISA kit is available for detecting anti-aMPV/C antibodies in ducks.

To bridge this diagnostic gap and eliminate the adverse effects of invasive sampling on egg production, we developed a non-invasive iELISA for detecting aMPV/C-specific IgY antibodies in duck egg yolks. This method is particularly advantageous for seroepidemiological surveillance and diagnosis in actively laying and breeding ducks, where sampling stress is a major concern.

## 2. Materials and Methods

### 2.1. Ethical Statement

This study was conducted in accordance with the Guidelines for the Welfare and Ethical Review of Laboratory Animals issued by the Ministry of Science and Technology of the People’s Republic of China. The experimental protocol was formally approved by the Laboratory Animal Management and Use Committee of the Fujian Academy of Agricultural Sciences (Approval No.: MYLISC2024-016).

### 2.2. Experimental Materials

The novel aMPV/C strain aMPV-FJ21 (10^6.56^ TCID_50_/mL), maintained in our laboratory, was used in this study [7]. Polyclonal IgY antibodies against H5 avian influenza virus (H5 AIV, strains H5-Re13, H5-Re15, and H5-Re14), Newcastle disease virus (NDV, strain La Sota), duck Tembusu virus (DTMUV, strain WF100), and duck plague virus (DPV, strain CVCC AV1222) were also obtained from our laboratory repository. Additionally, 151 clinical duck yolk samples were collected from ducks with egg drop or HFS on commercial farms in Fujian Province, China. These ducks had not been previously vaccinated against aMPV nor exposed to any aMPV subtype other than subtype C.

### 2.3. Antigen Preparation

To obtain sufficient virus stocks, Vero cells were inoculated with the novel aMPV/C strain aMPV-FJ21 (10^6.56^ TCID_50_/mL) and maintained in serum-free DMEM (Gibco, Grand Island, NE, US). The infected cells were harvested at 96 h post-infection (hpi) and stored at −70 °C. The harvested cells were subjected to three cycles of freezing and thawing, after which the virus was rendered non-infectious with 0.1% formaldehyde at 37 °C overnight. To confirm complete inactivation, the inactivated aMPV-FJ21 suspension was inoculated onto Vero cells. After 3 days, the supernatant was blind-passaged onto fresh Vero cells. The absence of CPE after blind passage indicated complete inactivation. Negative (DMEM only) and positive (live aMPV-FJ21) controls were included. For antigen purification, 300 mL of the inactivated suspension was clarified by centrifugation at 10,000× *g* for 15 min at 4 °C to remove cellular debris. Subsequently, the virus suspension was pelleted by ultracentrifugation at 45,000× *g* for 5 h. The concentrated virus was then resuspended in 1 mL of 0.01 M phosphate-buffered saline (PBS, pH 7.4) and used as the coating antigen for ELISA development and preparation of hyperimmune yolk antibody. Separately, the purified virus was characterized by transmission electron microscopy (TEM; H-7500, Hitachi, Japan) and BCA assay (Beyotime Biotechnology, Fuzhou, China).

### 2.4. Preparation of Hyperimmune Anti-aMPV/C Yolk Antibody

For the production of hyperimmune anti-aMPV/C IgY from egg yolks, 100 μg of the aforementioned antigen was thoroughly emulsified in an equal volume of Freund’s complete adjuvant (Sigma-Aldrich, Kenilworth, NJ, USA) at a 1:1 (*v*/*v*) ratio for primary immunization. Ten healthy 160-day-old laying Jinding ducks, confirmed to be seronegative for aMPV/C antibodies and negative for aMPV/C antigen, were then immunized via multiple subcutaneous injections in the dorsal region. For the second and third booster immunizations, 150 μg of antigen was emulsified in an equal volume of Freund’s incomplete adjuvant (Sigma-Aldrich, Kenilworth, NJ, USA) at a 1:1 (*v*/*v*) ratio. A final booster immunization was administered with 150 μg of antigen in the absence of adjuvant. Eggs were collected 3 days after the final immunization for downstream Anti-aMPV/C yolk antibody extraction. Ten ducks were mock-immunized with Vero cell lysate following the same protocol, and the resulting eggs were collected as negative yolk antibody samples. Throughout the experimental period, all ducks were housed in an isolated facility with ad libitum access to feed and water.

### 2.5. IgY Purification from Egg Yolks

IgY was purified from duck egg yolks using the chloroform–polyethylene glycol (PEG) precipitation method as previously described by Fishman et al. [27]. Briefly, duck eggs were surface-disinfected by soaking in 0.1% iodine solution for 1 min, and then yolks were carefully separated from albumen under aseptic conditions in a biosafety (Jinan Biobase Biotech Co., Ltd., Jinan, Shangdong, China). One milliliter of yolk was added to a centrifuge tube containing 2 mL of chloroform, followed by the addition of 1 mL of PBS and vigorous vortexing. The resulting mixture was then incubated at room temperature for 2 h. The aqueous phase was recovered by centrifugation at 5000 rpm for 10 min at 4 °C. PEG 6000 (Solarbio, Beijing, China) was added to the supernatant to a final concentration of 12% (*w*/*v*), and the solution was mixed thoroughly and kept at 4 °C overnight to allow complete precipitation. The precipitate was pelleted by centrifugation at 5000 rpm for 30 min at 4 °C, redissolved in PBS, and stored at −20 °C for subsequent use.

### 2.6. Establishment and Optimization of the iELISA for Detecting IgY in Egg Yolks

The optimal iELISA conditions were determined by checkerboard titration. Briefly, 96-well plates (Corning, New York, NY, USA) were coated with serially diluted aMPV-FJ21 antigen (0.15625 μg/mL, 0.3125 μg/mL, 0.625 μg/mL, 1.25 μg/mL, 2.5 μg/mL, 5 μg/mL) in carbonate buffer (CBS, 0.05 M, pH 9.6, Solarbio, Beijing, China) at 37 °C for 1 h, and different coating buffers (saline, PBS, CBS, and ddH_2_O) and conditions (4 °C overnight, 37 °C for 60 min, 120 min, or 150 min) were assessed. After washing three times, plates were blocked with various solutions (1% or 2% BSA, 5% or 10% skim milk, or 10% FBS) for 60 min~150 min at 37 °C. The optimal conditions were selected based on the highest P/N ratio.

For antibody incubation, the purified IgY (1:50, 1:100, 1:200, 1:400) and HRP-conjugated goat anti-duck IgG (1:2000, 1:4000, 1:8000, 1:16,000, KPL, Gaithersburg, MD, USA) were added sequentially, with incubation times optimized at 37 °C for 30 min, 60 min, 90 min, and 120 min. Finally, TMB substrate (Solarbio, Beijing, China) was added for 10 min, 15 min, 20 min, and 25 min, and OD_450nm_ was measured after termination. This optimization process systematically defined the ideal coating, blocking, antibody incubation, and substrate reaction conditions for the iELISA.

### 2.7. Determination of the Cut-Off Values

The diagnostic sensitivity, specificity, and cut-off value of the iELISA were all determined via receiver operating characteristic (ROC) curve analysis using duck egg yolk samples with IFA-verified infection status. Specifically, the cut-off value was defined as the threshold that yielded the maximum Youden index from the ROC curve, and the corresponding relative sensitivity and specificity were calculated accordingly. The area under the ROC curve (AUC), a metric reflecting the assay’s overall diagnostic accuracy, was computed. Diagnostic accuracy was graded into five tiers: non-informative (AUC ≤ 0.5), low accuracy (0.5 < AUC ≤ 0.7), moderate accuracy (0.7 < AUC ≤ 0.9), high accuracy (0.9 < AUC < 1), and perfect accuracy (AUC = 1). Statistical analyses were conducted with GraphPad Prism 8.0 (GraphPad Software, San Diego, CA, USA).

### 2.8. Evaluation of the Established iELISA

The specificity of the established iELISA was evaluated using positive IgY against AIV, NDV, DTMUV, and DPV. aMPV/C-positive and -negative IgY were used as controls.

To assess the repeatability of the iELISA, four aMPV/C-positive and four aMPV/C-negative IgY were randomly selected and tested in triplicate on both the same batch and different batches of ELISA plates. Variability is expressed as the coefficient of variation (CV), calculated as [standard deviation (SD)/mean] × 100% based on OD_450nm_ values of each sample group.

To evaluate iELISA sensitivity, three aMPV/C-positive IgY with distinct OD_450nm_ values were serially diluted twofold (1:200, 1:400, …, 1:12,800, and 1:25,600) and tested in parallel.

### 2.9. IFA

IFA was carried out following the protocol described in reference [7]. Briefly, Vero cells in 96-well plates were inoculated with aMPV-FJ21 at approximately 80% confluence. Following a 72 h incubation, the cells were fixed with 4% paraformaldehyde (Solarbio, Beijing, China) for 10 min at room temperature and blocked with 5% skim milk in PBS for 1 h at 37 °C. The cells were then incubated with suspected aMPV/C-specific IgY (1:100) overnight at 4 °C, washed three times, and subsequently incubated with Alexa Fluor 488-labeled goat anti-duck IgG (1:200, KPL, Gaithersburg, MD, USA) for 1 h at 37 °C. Finally, all samples were observed under a Nikon A1R confocal fluorescence microscope (Nikon Instruments, Inc., Melville, NY, USA). aMPV/C-negative and -positive IgY were used as controls.

### 2.10. Detection of aMPV/C-Specific IgY in Clinical Duck Egg Yolk Samples

A total of 151 egg yolk samples from ducks with egg production drops or HFS (80 Sheldrake ducks, 39 Muscovy ducks, and 32 Cherry Valley ducks) were collected from commercial duck farms and tested by the established iELISA. 46 of these samples were randomly selected for validation by IFA, and the concordance rate between the two methods was calculated.

## 3. Results

### 3.1. Verification of aMPV-FJ21 Inactivation

**Figure 1:**
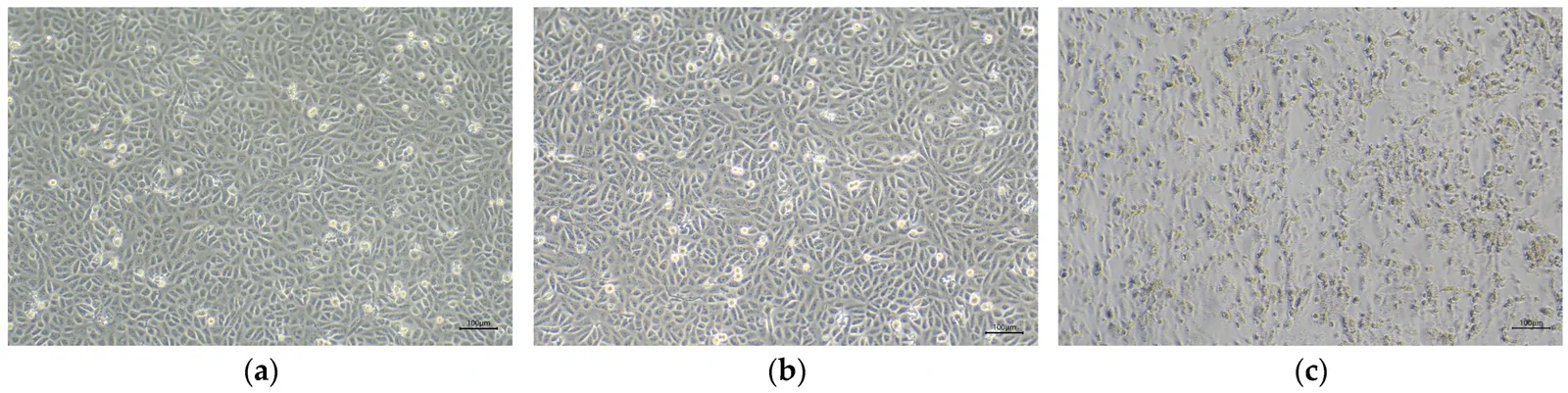
Verification of aMPV-FJ21 inactivation: (**a**) Normal Vero cells; (**b**) Vero cells inoculated with the inactivated aMPV-FJ21; (**c**) Vero cells inoculated with the live aMPV-FJ21.

Inactivation of aMPV-FJ21 was performed with 0.1% formaldehyde at 37 °C for 24 h. To confirm complete inactivation, the treated virus was inoculated onto Vero cell monolayers, and cytopathic effect (CPE) was observed for three days. No CPE was detected in cells inoculated with inactivated virus (Figure 1a) or in the medium-only negative control group (Figure 1b), whereas cells inoculated with the live virus showed extensive CPE including cell lysis, fragmentation, and detachment (Figure 1c).

### 3.2. Virus Purification and Identification

**Figure 2:**
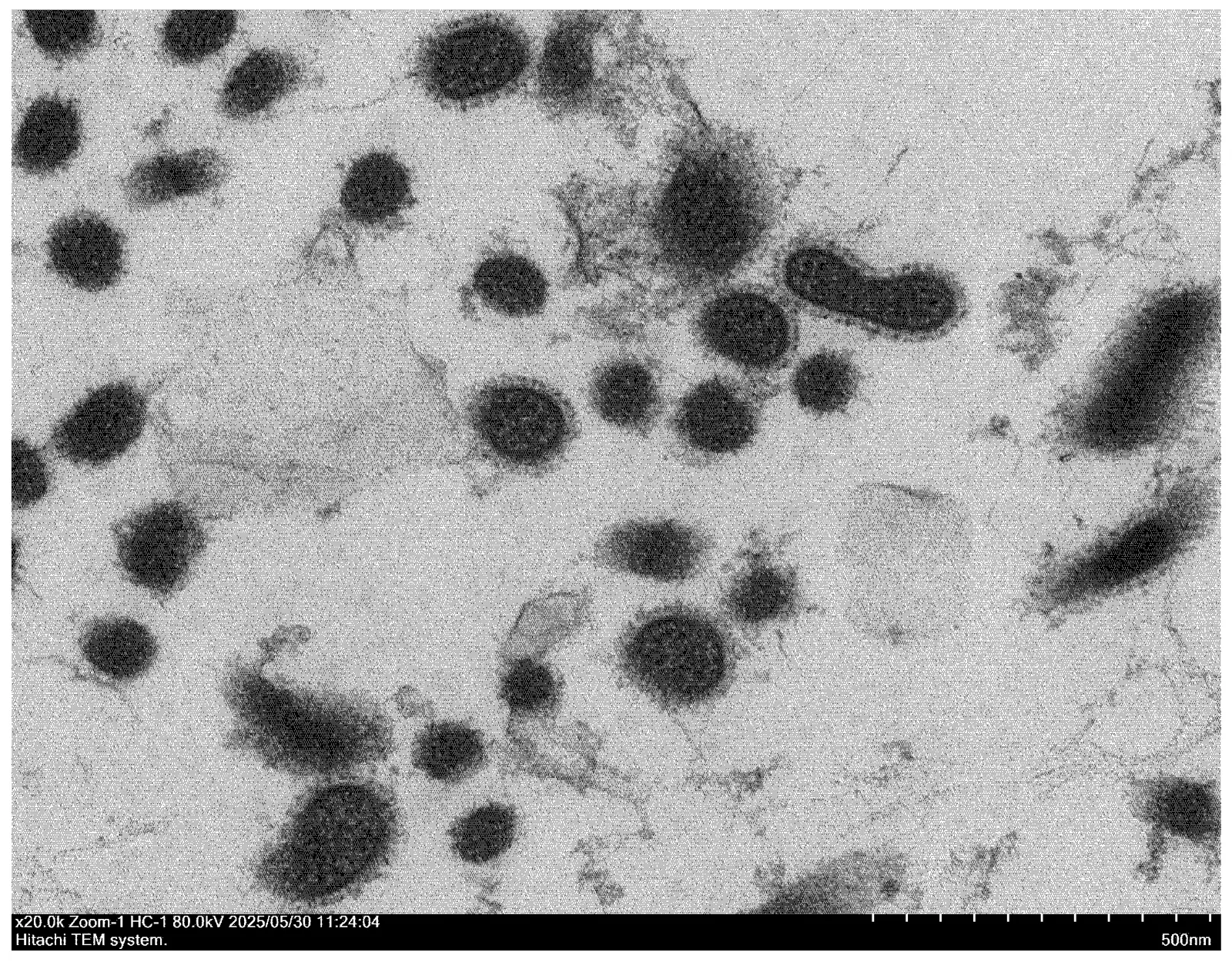
Transmission electron micrograph of purified aMPV-FJ21 virions. Spherical, irregular, and long filamentous particles, ranging from 20 to 500 nm in diameter, were observed.

Ultracentrifugation was employed to purify the aMPV-FJ21 strain, yielding a protein concentration of 1.85 mg/mL as determined by BCA. Examination of the entire microscopic field revealed that the virions were pleomorphic, displaying both spherical and irregular forms. This morphology was consistent with the characteristic appearance of aMPV/C, confirming the successful purification of intact virions (Figure 2).

### 3.3. Development of the iELISA for Detection of aMPV/C-Specific IgY in Duck Egg Yolks

**Figure 3:**
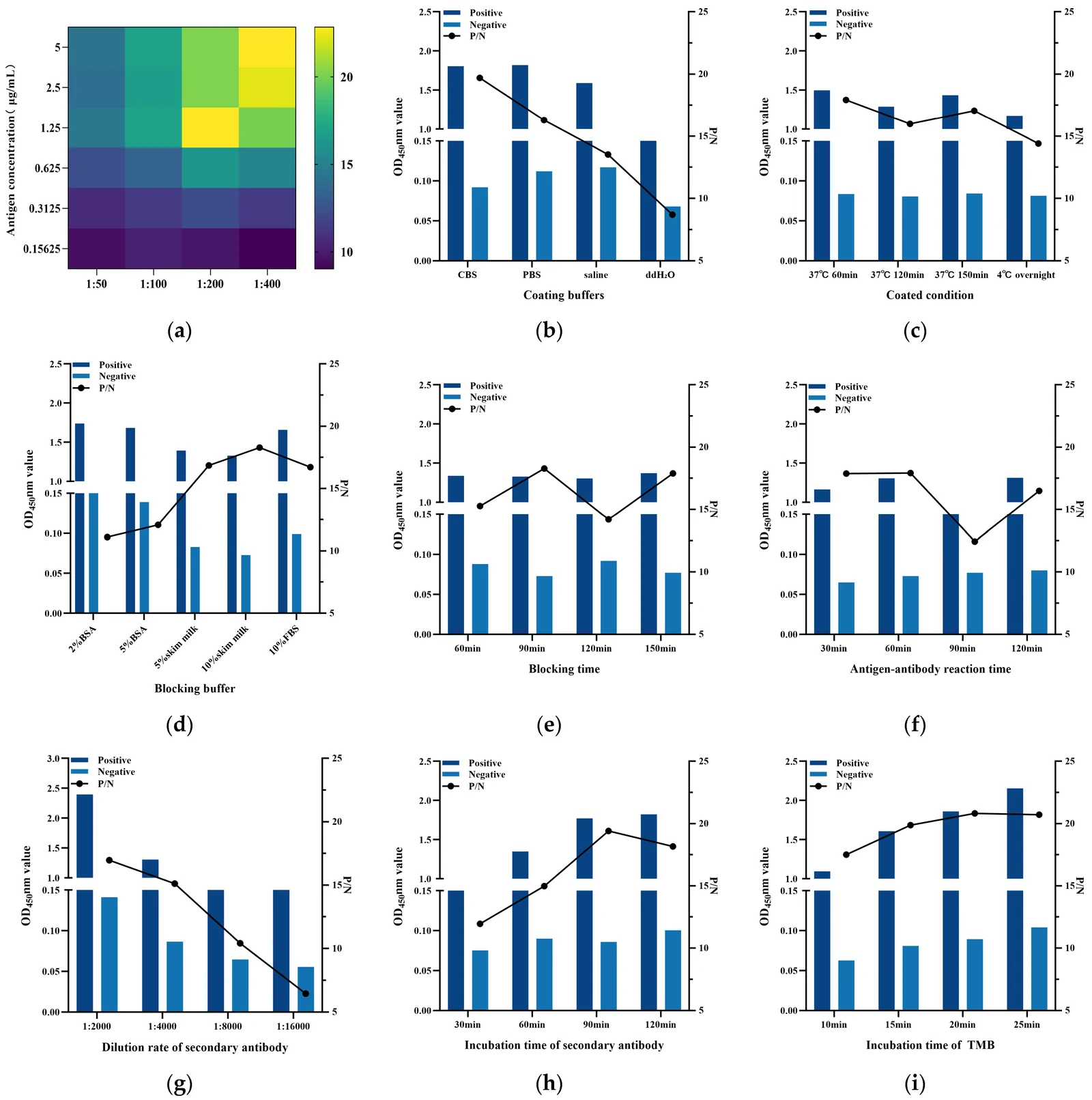
Development and optimization of an iELISA for detecting aMPV/C-specific IgY in duck egg yolks: (**a**) Determination of optimal antigen concentration and IgY dilution by checkerboard titration. (**b**,**c**) Evaluation of coating buffer and coating time. (**d**,**e**) Evaluation of blocking solution and blocking time. (**f**) Optimization of primary antibody (IgY) incubation time. (**g**,**h**) Optimization of secondary antibody dilution and incubation time. (**i**) Optimization of TMB substrate color development time.

An iELISA for the detection of aMPV/C antibodies was established, and all reaction conditions were systematically optimized. Checkerboard titration determined the optimal antigen coating concentration as 1.25 μg/mL and the optimal IgY dilution as 1:200 (Figure 3a). Further optimization identified the best conditions as follows: coating with CBS at 37 °C for 1 h (Figure 3b,c) and blocking with 10% FBS at 37 °C for 1.5 h (Figure 3d,e). Other optimal parameters were primary antibody incubation for 60 min (Figure 3f), secondary antibody (diluted 1:4000) incubation for 90 min (Figure 3g,h), and TMB substrate color development for 20 min (Figure 3i).

### 3.4. Cut-Off Values for the Self-Established iELISA

**Figure 4:**
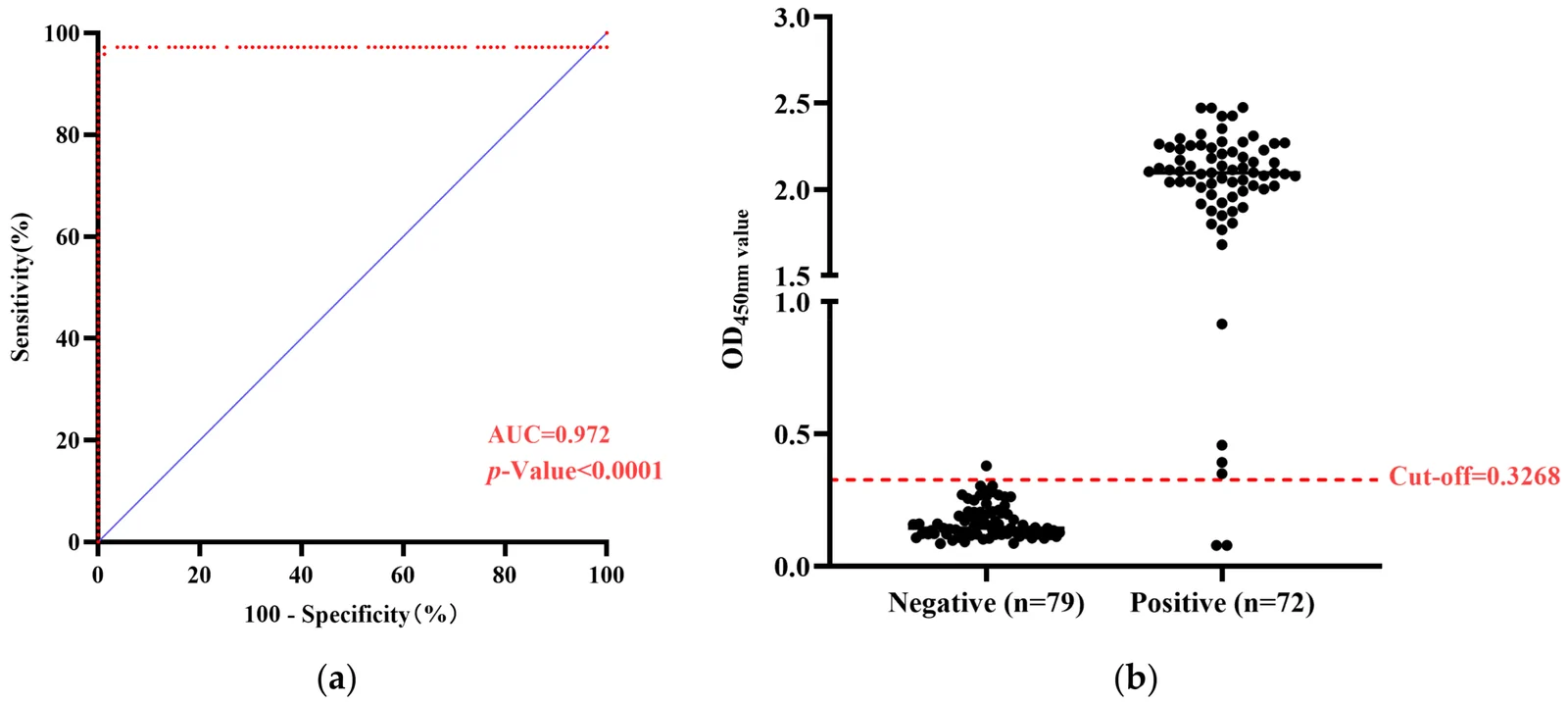
Determination of the cut-off value for the iELISA detecting aMPV/C-specific IgY in duck egg yolks: (**a**) ROC curve analysis for cut-off determination. The analysis was performed based on OD_450nm_ values from 72 aMPV/C-positive and 79 aMPV/C-negative egg yolk IgY, all previously identified by the IFA. Diagnostic accuracy levels are defined as non-informative (AUC ≤ 0.5), low accuracy (0.5 < AUC ≤ 0.7), moderate accuracy (0.7 < AUC ≤ 0.9), high accuracy (0.9 < AUC < 1), and perfect accuracy (AUC = 1). (**b**) Distribution of OD_450nm_ values across IFA-confirmed duck egg yolk sample groups. The *X*-axis represented two sample categories pre-validated by the IFA: aMPV/C-negative and aMPV/C-positive. The *Y*-axis showed the OD_450nm_ absorbance value of each sample tested by the iELISA. The red dashed horizontal line indicates the established optimal cut-off value of 0.3268.

The diagnostic performance of the iELISA was assessed with 72 aMPV/C-positive and 79 aMPV/C-negative IgY previously identified by IFA. ROC curve analysis determined the optimal cut-off value as 0.3268 (Figure 4a,b). At this threshold, the iELISA showed a diagnostic sensitivity of 97.22% (70/72) and a specificity of 98.73% (78/79), with a Youden index of 0.9595 (Table 1). The area under the AUC was 0.972 (95% CI: 0.934–1.000), indicating excellent diagnostic accuracy. Therefore, samples with OD_450nm_ ≥ 0.3268 were judged as positive, and those below the threshold were judged as negative.

### 3.5. Specificity, Reproducibility, and Sensitivity of the Self-Established iELISA

**Figure 5:**
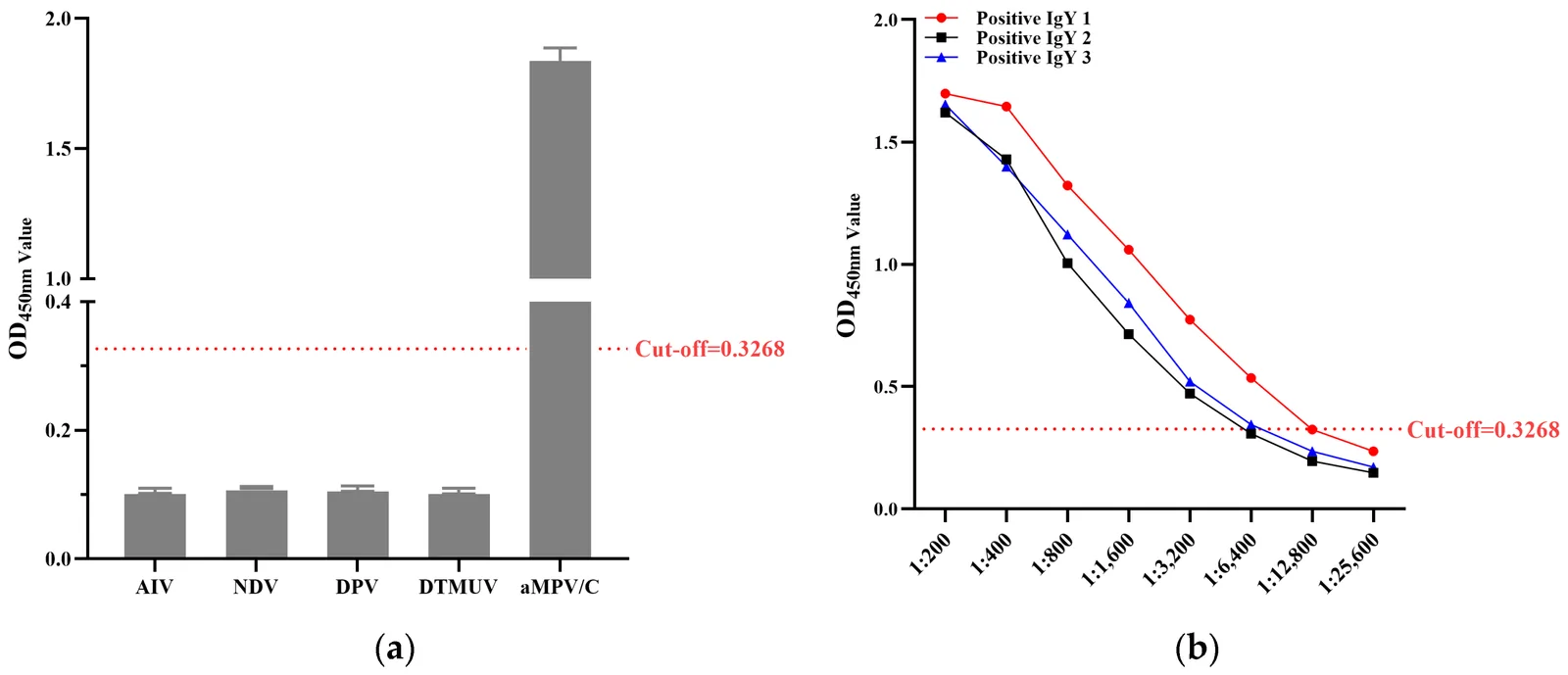
Analytical specificity and sensitivity of the self-established iELISA detecting aMPV/C-specific IgY in duck egg yolks: (**a**) Specificity evaluation using IgY positive against aMPV/C and other common duck pathogens (H5 AIV, NDV, DPV, and DTMUV). Only anti-aMPV/C-positive IgY samples were above the cut-off (red dashed line, 0.3268). (**b**) Sensitivity evaluation using two-fold serial dilutions of anti-aMPV/C-positive IgY (1:200 to 1:25,600). The iELISA remained positive up to a dilution of 1:3200.

The specificity of the iELISA was evaluated using IgY-positive egg yolk samples against other major duck pathogens, including AIV, NDV, DTMUV, and DPV. As shown in Figure 5a, only aMPV/C-positive samples yielded OD_450nm_ values above the cut-off, while all other pathogen-positive samples remained negative, confirming no cross-reactivity.

The reproducibility of the iELISA was evaluated using eight IgY antibodies with various OD_450nm_. The intra-assay and inter-assay CVs ranged from 0.80% to 3.32% and 0.81% to 5.26%, respectively (all <10%; Table 2), indicating high assay stability and precision.

The analytical sensitivity was determined by testing two-fold serial dilutions (1:200 to 1:25,600) of three anti-aMPV/C-positive IgY. The iELISA could stably detect specific antibodies up to a dilution of 1:3200 (Figure 5b), demonstrating high analytical sensitivity for aMPV/C antibody detection.

### 3.6. Comparison of the Self-Established iELISA and IFA

**Figure 6:**
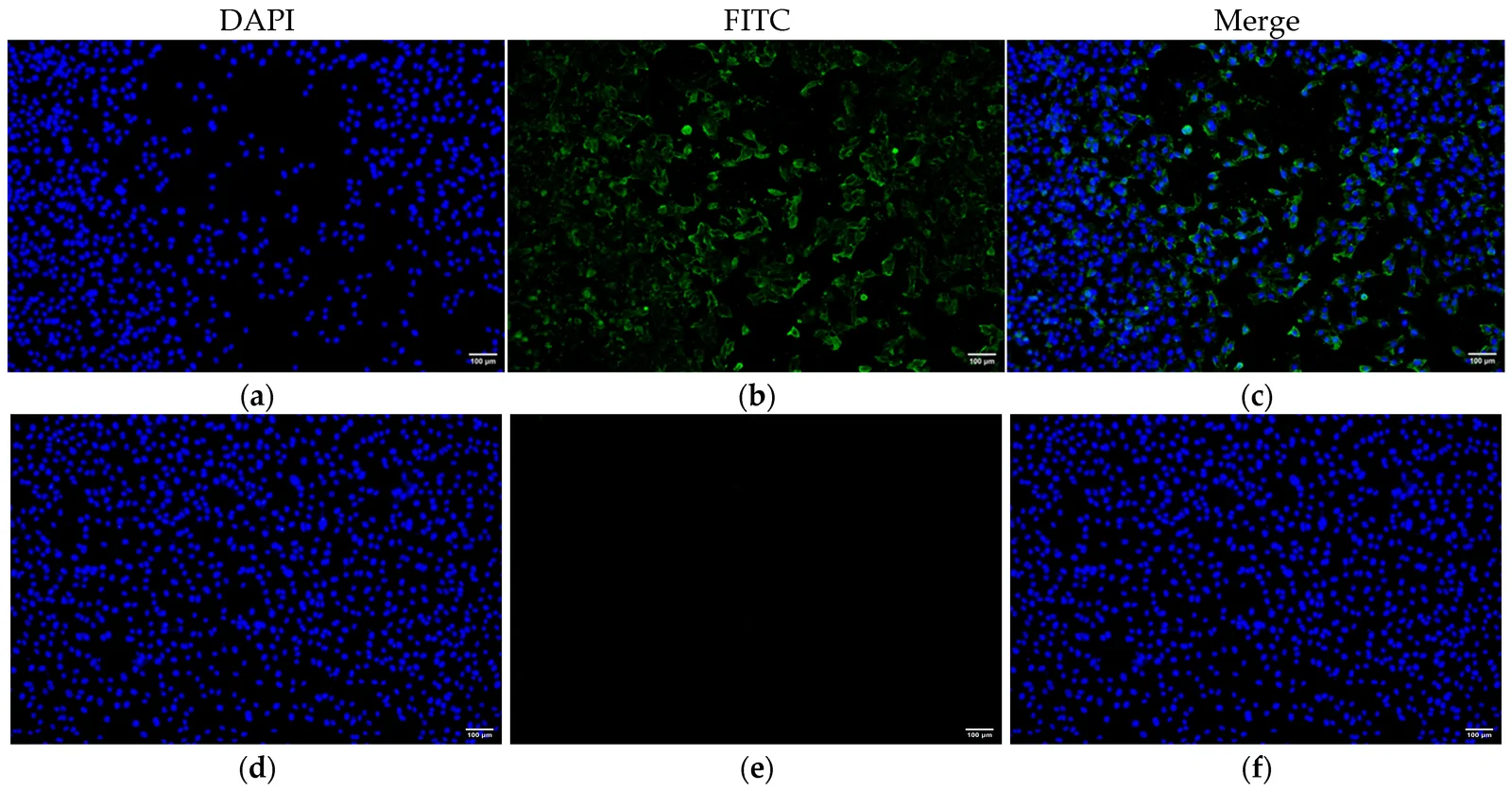
Detection of aMPV/C-specific IgY in duck egg yolks by IFA. (**a**–**c**) Vero cells infected with aMPV-FJ21 exhibited green fluorescence when incubated with anti-aMPV/C-positive IgY from duck egg yolk. (**d**–**f**) No green fluorescence signal was detected with anti-aMPV/C-negative IgY.

Clinical samples were collected from a duck farm in Fujian Province and detected by the established iELISA and IFA (Figure 6). The results (Table 3) showed that the positive rates of aMPV/C-specific IgY were 67.5% (56/80) in Sheldrake ducks, 53.8% (21/39) in Muscovy ducks, and 34.4% (11/32) in Cherry Valley ducks. The overall positive rate of aMPV/C-specific IgY was 58.3% (88/151). In addition, 46 samples were randomly selected for parallel detection, and the total coincidence rate between the two methods was 97.8% (45/46). These results indicate that the established iELISA is reliable and suitable for rapid clinical detection and field diagnosis (Table 4).

## 4. Discussion

The increasing prevalence of aMPV/C in Chinese duck populations and the emergence of a novel lineage linked to HFS have created an urgent need for duck-adapted diagnostic tools [7]. While several serological assays have been developed for aMPV detection in chickens and turkeys using recombinant structural proteins as coating antigens [24,27,28,29], no commercial ELISA is currently available for detecting aMPV/C antibodies in ducks. This diagnostic gap is partly due to the historical view that waterfowl were not primary hosts of the pathogen, which delayed the development of species-specific tools. To address this gap and overcome the limitations of invasive blood sampling in laying ducks, we developed a non-invasive iELISA based on inactivated, purified, novel aMPV/C virions for detecting aMPV/C-specific IgY antibodies in duck egg yolks.

The use of whole aMPV/C virions as the coating antigen offers distinct advantages over recombinant protein-based ELISAs. Recombinant proteins, despite their wide application, are often limited by incomplete epitope coverage, loss of conformational epitopes, and tag interference, all of which can compromise assay accuracy [30]. Comparative studies have consistently shown that whole-virus antigens provide superior sensitivity, particularly for detecting low-titer antibodies or early-stage antibodies post-infection [31]. For instance, virus lysate-based ELISAs exhibited higher sensitivity than peptide- and recombinant-based formats when tested with diluted positive samples [32]. In the context of aMPV detection, recombinant nucleocapsid (N) protein-based ELISAs have been established for chickens and turkeys [33], but their utility in ducks, especially for detecting antibodies against emerging variants, remains uncertain given the genetic divergence of the aMPV-FJ21 strain. In contrast, whole-virion antigens preserve a broad repertoire of conformational and linear epitopes, enabling detection of low-titer antibodies that may be missed by single-protein assays. This advantage is further supported by the robust propagation of the aMPV-FJ21 strain in Vero cells, which yielded substantially higher titers than previously reported at the same passage level [34,35]. High-titer virus stocks are critical for consistent whole-virion antigen preparation, ensuring sufficient antigen quantity and quality for large-scale ELISA production. The use of a single master seed stock and serum-free culture conditions further ensured batch-to-batch reproducibility and minimized exogenous protein interference, both essential for translation of the assay into routine diagnostic use. In this study, each batch of purified virus was quantified by BCA assay (inter-batch CV < 5%) to ensure consistent protein concentration, and the purity and integrity of the viral particles were verified by TEM. For future studies, each batch of purified virus should be further verified by SDS-PAGE or Western blot to confirm antigenic epitope integrity.

Epidemiological application of the established iELISA revealed an overall seroprevalence of 58.3% across 151 laying ducks, with notable breed-dependent differences: Sheldrake ducks had the highest positivity rate (67.5%), followed by Muscovy ducks (53.8%) and Cherry Valley ducks (34.4%). These findings confirm the widespread circulation of aMPV/C in domestic breeder and layer duck populations in China [14,15,16] and suggest that breed-related susceptibility may contribute to the observed differences in viral prevalence. The higher seroprevalence in indigenous breeds such as Sheldrake and Muscovy ducks warrants further investigation into host genetic factors and immune response profiles that may influence aMPV/C susceptibility. Our findings align with global reports of aMPV/C infection in wild waterfowl: Notably, 22% of ducks tested positive for aMPV across multiple lakes in Ontario, Canada [36], and aMPV/C was detected in mallard flocks and a Eurasian Wigeon in Italy [37,38], suggesting that wild waterfowl may act as a reservoir for viral dissemination. The breed-dependent differences observed in this study may also reflect varying levels of field exposure, management practices, or genetic resistance among breeds, all of which require further investigation.

The agreement (97.8%) between the iELISA and IFA across 46 randomly selected samples supports the reliability of our assay for large-scale serological surveillance. The single discordant case (IFA-negative but iELISA-positive) was most likely due to the higher analytical sensitivity of ELISA, which can detect low-titer antibodies undetectable by the less sensitive IFA method. This enhanced sensitivity is particularly valuable for early detection of subclinical infections, which is critical for timely intervention and disease control in breeder duck flocks. Given that aMPV/C infection causes significant economic losses via reduced egg production, with affected flocks experiencing egg drop rates of 10–30% [4,6,7], early detection of subclinical infections enables prompt implementation of biosecurity measures and vaccination strategies before clinical outbreaks occur. The high sensitivity of the iELISA also makes it suitable for monitoring vaccination efficacy and conducting large-scale epidemiological surveys to track the spread of aMPV/C across different regions and duck breeds in China.

From a practical perspective, the use of egg yolks instead of blood samples offers substantial advantages for field application, addressing key limitations of conventional serological testing in laying duck populations. Firstly, it eliminates the stress associated with bird handling and venous blood collection. Blood sampling requires physical restraint and venipuncture, which induces acute stress in ducks and can transiently reduce egg production, a critical concern for commercial laying and breeder flocks already at risk of egg drop from aMPV/C infection [39]. In contrast, egg sampling requires no additional animal handling beyond routine egg collection, avoiding stress-related production losses entirely. Each yolk IgY assay consumes one duck egg, yet this loss is minimal relative to the egg production decline caused by blood collection stress (significant drop of ≥10%). Secondly, it greatly reduces labor and operational costs. Blood collection typically requires trained veterinary personnel and is time-consuming, especially for large flocks. Egg sampling can be performed by on-farm staff with minimal training, and dozens of samples can be collected rapidly during daily egg collection routines. Thirdly, it improves animal welfare and biosecurity: the method avoids invasive procedures, aligning with the 3R principles for animal research, and eliminates the risk of cross-infection via contaminated needles or equipment during blood sampling [40,41]. Finally, egg yolk sampling is highly amenable to longitudinal surveillance [41]. Unlike blood sampling, which is disruptive and difficult to repeat frequently in the same flock, egg samples can be collected continuously over time to monitor infection dynamics, antibody kinetics, and vaccine-induced immunity without disrupting farm operations. These practical benefits make the iELISA particularly well-suited for routine surveillance and disease control programs under field conditions.

Limitations of this study should be acknowledged. The field samples were collected from a limited number of provinces in China. The generalizability of the assay thus requires further validation across broader geographical regions and diverse production systems. We will carry out a nationwide seroepidemiological survey of laying breeder ducks across China to systematically delineate the epidemiological prevalence pattern of aMPV/C. Additionally, while whole-virion antigens prepared via cell culture and purification incur higher costs than prokaryotically expressed recombinant proteins, the required antigen dosage is relatively modest, rendering the overall cost acceptable for routine diagnostic and surveillance applications.

## 5. Conclusions

In conclusion, we first developed and validated a non-invasive iELISA for the high-throughput detection of aMPV/C-specific IgY in laying ducks. The assay exhibited excellent specificity, sensitivity, and repeatability, making it suitable for large-scale serological surveillance in laying ducks. Furthermore, our findings confirmed that aMPV/C infection is present in both laying meat-type breeder ducks and layer-type duck populations in China.

## Figures and Tables

**Table 1:** Optimal cut-off value of the self-established iELISA detecting aMPV/C-specific IgY determined by the Youden index.

Cut-Off Value	Sensitivity%	Specificity%	Youden Index
0.3268	97.22	98.73	0.9595
0.3861	95.83	100	0.9583
0.3647	95.83	98.73	0.9456
0.4255	94.44	100	0.9444
0.2943	97.22	96.2	0.9342

**Table 2:** Repeatability assessment of the self-established iELISA detecting aMPV/C-specific IgY in duck egg yolks.

Duck Egg Yolk Samples	Intra-Assay		Inter-Assay	
	Mean ± SD	CV (%)	Mean ± SD	CV (%)
Positive IgY 1	2.291 ± 0.052	2.26	2.282 ± 0.053	2.33
Positive IgY 2	2.246 ± 0.020	0.91	2.240 ± 0.037	1.66
Positive IgY 3	1.915 ± 0.075	3.90	1.914 ± 0.015	0.80
Positive IgY 4	2.351 ± 0.088	3.73	2.375 ± 0.020	0.86
Negative IgY 1	0.113 ± 0.006	5.26	0.112 ± 0.003	2.53
Negative IgY 2	0.155 ± 0.001	0.81	0.151 ± 0.003	2.05
Negative IgY 3	0.170 ± 0.008	4.46	0.166 ± 0.006	3.32
Negative IgY 4	0.131 ± 0.005	4.29	0.130 ± 0.001	1.06

**Table 3:** Detection results of clinical samples by the self-established iELISA for detecting aMPV/C-specific IgY in duck egg yolks.

Duck Breeds	No. of Tested Samples	No. of Positive Samples	Positive Rate (%)
Sheldrake duck	80	56	67.5
Muscovy duck	39	21	53.8
Cherry Valley duck	32	11	34.4
Total	151	88	58.3

**Table 4:** Comparison of the self-established iELISA and IFA for detecting aMPV/C-specific IgY in duck egg yolks.

Duck Egg Yolk Samples	Self-Established iELISA		
	Positive Samples	Negative Samples	Total
Positive samples tested by IFA	35	0	35
Negative samples tested by IFA	1	10	11
Total	36	10	46
Coincidence rate	100%	90.9%	97.8%

## Data Availability

The original contributions presented in this study are included in the article. Further inquiries can be directed to the corresponding authors.

## Abbreviations

The following abbreviations are used in this manuscript:

aMPV/C	Avian metapneumovirus subtype C
AIV	Avian influenza virus
AUC	Area under the ROC curve
CV	Coefficient of variation
DTMUV	Duck Tembusu virus
DPV	Duck plague virus
iELISA	Indirect enzyme-linked immunosorbent assay
IgY	Immunoglobulin Y
HFS	Hydrosalpinx fluid syndrome
hpi	Hour post-infection
NDV	Newcastle disease virus
PEG	Chloroform-polyethylene glycol
ROC	Operating characteristic
SD	Standard deviation
TEM	Transmission electron microscopy
